# Clinical Applicability and Cutoff Values for an Unstructured Neuropsychological Assessment Protocol for Older Adults with Low Formal Education

**DOI:** 10.1371/journal.pone.0073167

**Published:** 2013-09-16

**Authors:** Jonas Jardim de Paula, Laiss Bertola, Rafaela Teixeira Ávila, Lafaiete Moreira, Gabriel Coutinho, Edgar Nunes de Moraes, Maria Aparecida Camargos Bicalho, Rodrigo Nicolato, Breno Satler Diniz, Leandro Fernandes Malloy-Diniz

**Affiliations:** 1 Laboratory of Neuropsychological Investigations (LIN), Universidade Federal de Minas Gerais, Belo Horizonte, Minas Gerais, Brazil; 2 INCT de Medicina Molecular, Faculdade de Medicina, Universidade Federal de Minas Gerais, Belo Horizonte, Minas Gerais, Brazil; 3 Clinical Staff, D'Or Institute for Research and Education (ID'Or), Rio de Janeiro, Rio de Janeiro, Brazil; 4 Programa de Pós-graduação em Ciências Morfológicas, Universidade Federal do Rio de Janeiro, Rio de Janeiro, Rio de Janeiro, Brazil; 5 Departamento de Clínica Médica, Faculdade de Medicina, Universidade Federal de Minas Gerais, Belo Horizonte, Brazil; 6 Departamento de Saúde Mental, Faculdade de Medicina, Universidade Federal de Minas Gerais, Belo Horizonte, Minas Gerais, Brazil; University of Manchester, United Kingdom

## Abstract

**Background and Objectives:**

The neuropsychological exam plays a central role in the assessment of elderly patients with cognitive complaints. It is particularly relevant to differentiate patients with mild dementia from those subjects with mild cognitive impairment. Formal education is a critical factor in neuropsychological performance; however, there are few studies that evaluated the psychometric properties, especially criterion related validity, neuropsychological tests for patients with low formal education. The present study aims to investigate the validity of an unstructured neuropsychological assessment protocol for this population and develop cutoff values for clinical use.

**Methods and Results:**

A protocol composed by the Rey-Auditory Verbal Learning Test, Frontal Assessment Battery, Category and Letter Fluency, Stick Design Test, Clock Drawing Test, Digit Span, Token Test and TN-LIN was administered to 274 older adults (96 normal aging, 85 mild cognitive impairment and 93 mild Alzheimer`s disease) with predominantly low formal education. Factor analysis showed a four factor structure related to Executive Functions, Language/Semantic Memory, Episodic Memory and Visuospatial Abilities, accounting for 65% of explained variance. Most of the tests showed a good sensitivity and specificity to differentiate the diagnostic groups. The neuropsychological protocol showed a significant ecological validity as 3 of the cognitive factors explained 31% of the variance on Instrumental Activities of Daily Living.

**Conclusion:**

The study presents evidence of the construct, criteria and ecological validity for this protocol. The neuropsychological tests and the proposed cutoff values might be used for the clinical assessment of older adults with low formal education.

## Introduction

The neuropsychological evaluation plays an important role in the differential diagnostic between normal and pathological aging cognitive processes [1]. It is of particular relevance to differentiate subjects with mild cognitive impairment (MCI) from those in the early stages of dementia, e.g. mild Alzheimer's disease (AD), when cognitive screening tests are not sensitive for the differential diagnosis [2]. The neuropsychological evaluation usually presents similar or greater sensitivity and specificity for the identification of AD versus MCI when compared to other diagnostic procedures. For instance, in a recent study the neuropsychological assessment showed higher accuracy (84%) to differentiate MCI from AD patients, followed by structural Magnetic Resonance Imaging (82%), PET-FDG (76%), and cerebrospinal fluid biomarkers (73%) [3]. The combinations of different procedures increase the diagnosis accuracy, although the estimated additional gain in effect sizes were small.

Formal education has a major impact in the performance on cognitive tests and can bias the interpretation of test results. Educational level influences the performance on cognitive screening test, such as the Mini-Mental State Examination and the Category Fluency Test, and cut-off scores for dementia diagnosis based on the educational level have been widely used [4], [5]. The performance on structured cognitive assessment batteries for diagnosis of dementia, such as the Mattis Dementia Rating Scale [6], the CERAD Battery [7], and the CAMCOG [8] is also influenced by educational level. Educational attainment can also affect the performance on neuropsychological tests that evaluate specific cognitive domains. Previous studies showed that the performance on several neuropsychological tests designed to assess language, episodic memory, and executive function is significantly biased by education [1], [9]–[13]. In developing countries, this is a very important issue, given the high proportion of older adults with no or few years of formal education [14]. It is, therefore, of utmost importance to develop and adapt neuropsychological batteries taking into account the effect of formal education to reduce the risk of bias and misclassification of subjects.

In clinical neuropsychology the use of an unstructured assessment protocol allows the clinician to carefully choose the neuropsychological tests according to a cognitive model and his clinical hypothesis, mapping different cognitive domains in a comprehensive way [1]. However, there few studies evaluating the psychometric properties of neuropsychological instruments for older adults with low formal education. The present study aims to investigate the psychometric properties of a neuropsychological assessment protocol designed for the evaluation of older adults with low educational level. We assessed its factor structure, criterion-related validity and ecological validity. In addition, we proposed cut-off scores to discriminate the diagnostic groups (AD, MCI and normal aging).

## Materials and Methods

### Participant's recruitment and assessment

In the present study 274 consecutive older adults were assessed. Participants were enrolled in an ongoing work evaluating the relationship of depression and dementia in the elderly. The participants or caregivers who did not show interest in joining the study were referred and treated normally by the patient service center, not suffering any burden with the non-participation. This study was approved by the local Ethics Committee (registry 334/06) and carried out in accordance with the Helsinki declaration. All the participants (and caregivers for patients with suspected dementia) gave written consent for the participation. Participants with less than 60 years, previous history of neurological or psychiatric disorders (except for depression), use of typical and atypical antipsychotics, evidence of major vascular events on brain computed tomography scans, severe sensorial or motor impairments or other clinical conditions which may influence the neuropsychological performance (such as hypothyroidism or B12 vitamin depletion), history of alcohol or other substance abuse and patients which a close caregiver was not present on the assessment were excluded from the present study.

All subjects underwent a comprehensive gerontological evaluation which included the cognitive assessment with the administration of the Mattis Dementia Rating Scale [6], the Mini-Mental State Examination [4], the Verbal Learning test of the CERAD Neuropsychological Battery [7], the Clinical Dementia Rating [15]. Depressive symptoms were assessed by the Short-Version of the Geriatric Depression Scale (GDS-15) [16]. Formal education assessed in the initial clinical interview was reported in years, discounting repetitions. Participants with less than one year and unable to read or write simple sentences were considerate illiterates. Participants which, do not have formal schooling, but were able to read and write were classified with one year of formal education.

The performance on neuropsychological tests used for diagnosis was interpreted based on cut-off values for the Mini-Mental State Examination (18 points for illiterate, 23 points for participants with 1 to 7 years of formal education and 26 for participants with 8 years or more). For the Mattis Dementia Rating total score and Subscales the “-2 Standard-Deviations” guideline (based on normative values stratified by education) was used. For the CERAD Neuropsychological Battery, we adopted as cut-off the first quartile stratified by education, following Nitrini and colleagues [7] recommendations. The procedure stratifies formal education in Illiterate, Low Educated Literate (less than 4 years of formal education) or Standard Educated Literate (4 or more years of formal education). The lower quartile has the following values: Immediate recall (Illiterate  = 3, Low Educated Literate  = 4, Standard Educated Literate  = 5), Delayed Recall (Illiterate  = 3, Low Educated Literate  = 4, Standard Educated Literate  = 4).

Neurocognitive status was adjudicated at expert multidisciplinary meetings, taking into account all clinical, cognitive assessment, laboratorial, and neuroimaging data when available. Functional status was investigated based on caregiver's reports about activities of daily living and by the functional components of the Clinical Dementia Rating [15]. The performance on cognitive tests was adjusted for age and educational status, based on Brazilian norms for each test. Alzheimer's disease was diagnosed according to the NINCDS-ADRDA [17] guidelines. The diagnosis of MCI was made according to Mayo Clinic criteria [18] as follows: 1) subjective cognitive complaint, preferably corroborated by an informant; 2) objective impairment in the performance on cognitive tests of the CERAD Neuropsychological Battery and on the Mattis Dementia Rating Scale, but not severe enough to reach dementia diagnosis; 3) preserved global cognitive function (Mini-Mental State Examination scores above the cutoff for dementia based on formal education); 4) preserved or minimal impairments in activities of daily living 5) not demented.

A total of 85 subjects were identified as MCI and 93 with AD. Ninety-six subjects with no evidence of cognitive impairment were included as a comparison group (“normal cognitive aging – NA” group). Normal aging and MCI participants had MMSE scores above the cutoff for dementia according to formal education [4]. Considering the cutoff 5/6 (case/non case) on the Geriatric Depression Scale, 35% of the NA, 26% of MCI and 29% of AD participants has significant depressive symptoms.

### Neuropsychological Assessment

The protocol used in the present study was designed to assess episodic memory, attention, executive functions, visuospatial abilities and language. It was designed to be fully administered in one session of 90 minutes, increasing its usefulness in the clinical contexts where the time and human resources for the neuropsychological assessment are scarce. The tests were selected based on Brazilian studies which investigated their psychometric properties on older adults with low formal education. Two neuropsychologists (LFM-D and JJP) searched the Brazilian and international literature for neuropsychological tests which: 1) may be suitable for older adults with low formal education (e.g.: avoiding reading or calculation, complex drawing and fine motor coordination); 2) have previous studies reporting, at least partially, validity for this population, 3) could be all applied on a 90 minutes assessment section, 4) tests did not have copyright agreements, with stimuli and application procedures freely available. The following tests were selected:

*Rey Auditory Verbal Learning Test (RAVLT)*: a measure of episodic memory containing five learning trials of a 15 word list followed by a distractor, an immediate recall (RAVLT IR), a 25-minutes delayed recall (RAVLT DR) and a recognition Trial (RAVLT Rec). The Brazilian version of the test proposed by Malloy-Diniz and colleagues [9] and validated for older adults [11] was chosen for the present study. The RAVLT has good criterion validity for the diagnosis of neurocognitive disorders in older adults, such as AD, MCI and major depression, according to a recent review [19].

*Frontal Assessment Battery*: designed as a bedside screening test for frontal-executive impairment [20], this test is a brief and well validated screening test for older adults, assessing different executive components by six subtests (similarities, fluency, motor planning, selective attention, inhibitory control and environmental autonomy). One of the Brazilian versions was used and the total score adopted as variable, since it was previously validated and had adequate normative values [21]. The test performance is usually compromised in clinical conditions related to frontostriatal dysfunction, such as Frontotemporal dementia, Progressive Supranuclear Palsy and Parkinson's disease [20]. The battery shows moderate correlations with classical executive-functions tests [20]–[23].

*Short version of the Token Test*: this language comprehension test designed for the detection of aphasia involves verbal orders of increasing difficulty (input) and motor sequences on colored tokens (output). The short version (36 items) is adapted [24], validated [10] and has adequate normative data [13] for this population. The test was divided in two components for this study, based on the factor structure proposed on a previous report [10]: Token Test – Attention (items 1 to 15) and Token Test – Comprehension (items 16 to 36).

*Category Fluency* and *Letter Fluency*: The verbal fluency tests are classical screening tests for cognitive impairment, usually associated with the executive functions. Two categories and one letter were chosen for the protocol, based on the normative and validity studies for the Brazilian population: *Animals* [5], *Fruits* [12] and “*S”* [25] generated in one minute. The test is very sensitive for cognitive impairment in different clinical conditions [2], [10], [12].

*Digit Span*: a classical measure of verbal working memory, a cognitive process related to storage and manipulation of verbal information. Kessels, van den Berg, Ruis and Brands [26] suggest the use of a product score between the maximum span and the number of correct trials (2 per span) as a more general measure of working memory efficiency. A previous report validated this method for older adults with low formal education [22]. In AD, the phonological loop of the working memory (assessed by the Digit Span Forward) is usually preserved, while the executive components (assessed by Digit Span Backward) are slightly impaired [27].

*Clock Drawing Test*: A classical drawing task designed for the assessment of cognitive impairment in older adults and a widely used test for cognitive screening. The Shulman's [28] (0 to 5 points, higher scores represent better performance, pre-drawn circle). This version was chosen for the present study, since it's one of the most sensitive for neurocognitive disorders in the elderly [29].

*Stick Design Test*: a test used for the assessment of visuospatial abilities consisted of four bi-dimensional models where the subject must reproduce them using four matches [30]. The models differ in global configuration (open and closed models), alignments, angles and orientation of match heads. The test is a good alternative to drawing tasks, since for very low educated or illiterate individuals the more traditional constructional praxis tests may cause negative emotional reactions and very low scores [31]. The Stick Design Test shows good criterion validity for dementia, even superior to drawing tests in patients with very low formal education [32].

*The Neuropsychological Investigations Laboratory Naming Test* (TN-LIN): developed as a measure of naming abilities in children and older adults with low formal education [33]. Based on classical naming paradigms, the TN-LIN uses 65 black-white line drawings divided into nouns (40), verbs (10) and professions (15). The nouns are divided in objects (15), animals (10), food (5), transports (4), and clothes (5). The nouns (TN-LIN Nouns), verbs (TN-LIN Verbs) and professions (TN-LIN Professions) were used for further analysis.

*Activities of Daily Living:* we used the Basic Daily Life Activities Index and the Instrumental Daily Life Activities Index, based on the Katz [34] and Lawton [35] indexes respectively, to assess functional performance. Each activity of daily living was scored, based on information provided by a close caretaker, as “2” (no functional impairment), “1” (partial dependence of human help in performing the daily life activity) and “0” (complete dependence of human help on performing the daily life activity). Scores for basic activities range from 0–12 and instrumental activities from 0–16. The general score (0–28 points) was used in this study. Lower scores indicated greater impairment.

### Statistical procedures

Performance on neuropsychological assessment tests and socio-demographic characteristics were assessed by one-way ANOVA, and the Sidak's post hoc test was used to assess pairwise group differences. Effect sizes for this analysis were computed by the eta-squared. Keeping in mind the hierarchical structure of the cognitive system [36], which leads to significant associations among neuropsychological measures, a principal axis factoring and an oblique (direct oblimin [37]) rotation were adopted, allowing the encountered factors to correlate. The criteria used for components formation include eigenvalues greater than 1 and convergent scree-plot analysis by two independent judges. The Keiser-Meyer-Olkim (KMO) test of sampling adequacy and the Bartlett's test of sphericity were used to assess the viability of the factor extraction. Cronbach's alpha of each factor was computed for the assessment of reliability. These procedures aims to assess if the proposed battery keeps its construct validity for the assessment of Executive Functions, Language, Memory and Visuospatial Abilities on the studied population, assuring it's clinical applicability. Another advantage of factor analysis is the reduction of the amount of test variables, reducing the probability of a Type 1 error on further analysis.

The ecological validity of the neuropsychological assessment was investigated by linear regression models, containing the ADL measures as dependent variables and the components of the factor analysis (extracted by the regression method and standardized based on the non-depressed normal aging participants scores) as predictors. We adopted stepwise procedures to reduce multicollinearity.

We carried out Receiver Operator Characteristic (ROC) curve analyses to compare the performance of each neuropsychological test to differentiate the diagnostic groups. Cutoffs for clinical use considering the best ration between sensitivity and specificity were calculated. Finally, we performed a multinomial logistic regression analysis, with diagnosis as dependent variable and the neuropsychological tests as independent variables; to evaluate which neuropsychological tests best differentiate the diagnostic groups. To reduce multicollinearity, we adopted a stepwise procedure (*forward entry*, entering criteria: 0.05 and exclusion criteria 0.10).

For the criterion related validity procedures, only participants with GDS-15 scores below 6 were selected. The Mini-Mental State Examination total score was included in the models, since the neuropsychological measures should have an addictive power with this screening measure for the patient's diagnosis. All the statistical procedures were performed on SPSS 19.0 (Chicago, IL) and statistical significance was set at α<5%.

## Results

The three groups did not differ in clinical and socio-demographic and clinical characteristics. As expected, we found significant differences in the scores of all neuropsychological tests among diagnostic groups, with effect sizes ranging from small to large (Table 1).

**Table 1:** Participants description, neuropsychological assessment and group comparisons for the whole sample.

Sociodemographic and neuropsychological assessment	NA (1)N = 96, F = 64 Dep = 34		MCI (2)N = 85. F = 51 Dep = 22		Mild AD (3)N = 93, F = 51 Dep = 27		Group Comparison's		
	M	SD	M	SD	M	SD	F	η^2^	Sidak's
Age	72.61	7.76	73.18	8.46	74.57	6.65	1.63	-	-
Education	5.22	4.29	4.71	4.00	4.82	3.46	0.43	-	-
Geriatric Depression Scale [Max = 15]	4.33	3.95	2.94	2.84	3.83	3.22	3.86*	0.03	1 = 3<2
Mini-Mental State Examination [Max = 30]	25.75	3.85	23.52	3.62	20.59	3.98	43.12**	0.24	1>2>3
Frontal Assessment Battery [Max = 18]	13.57	3.33	11.82	2.86	9.06	3.22	48.95**	0.27	1>2>3
Category Fluency (Animals)	13.99	4.70	11.14	3.39	8.48	3.82	44.05**	0.25	1>2>3
Category Fluency (Fruits)	11.96	3.94	9.36	2.36	7.85	2.71	42.28**	0.24	1>2>3
Letter Fluency (S)	9.99	4.32	9.15	4.00	7.11	3.44	13.28**	0.09	1 = 2>3
Digit Span Forward [Max = 144]	34.04	18.05	34.48	14.07	28.90	14.74	3.54*	0.03	1 = 2>3
Digit Span Backward [Max = 144]	15.60	9.97	12.51	10.16	8.98	7.45	12.11**	0.08	1 = 2>3
Stick Design Test [Max = 12]	11.66	0.90	11.08	1.81	10.27	2.43	13.85**	0.09	1 = 2>3
Clock Drawing Test [Max = 5]	3.53	1.78	2.66	1.74	2.01	1.60	18.69**	0.12	1>2>3
RAVLT – A1 [Max = 15]	4.59	1.75	3.32	1.30	2.84	1.41	33.94**	0.20	1>2 = 3
RAVLT – IR [Max = 15]	6.44	3.30	3.48	2.32	2.18	1.95	66.53**	0.33	1>2>3
RAVLT – DR [Max = 15]	6.55	3.55	3.21	2.53	1.87	1.83	72.83**	0.35	1>2>3
RAVLT – Rec [Max = 15]	7.78	4.45	0.78	6.64	−1.34	6.27	63.31**	0.32	1>2>3
RAVLT – Total [Max = 75]	35.07	11.81	26.32	8.72	21.40	7.47	49.66**	0.27	1>2>3
TN-LIN (Nouns) [Max = 40]	37.22	2.78	36.82	2.50	33.66	3.93	35.81**	0.21	1 = 2>3
TN-LIN (Verbs) [Max = 10]	9.57	1.03	9.66	0.72	8.92	1.46	11.70**	0.08	1 = 2>3
TN-LIN (Professions) [Max = 15]	13.38	2.56	13.08	1.85	11.28	3.13	17.90**	0.12	1 = 2>3
Token Test – Attentional [Max = 15]	14.74	0.67	14.59	0.80	14.33	1.09	5.44*	0.04	1 = 2>3
Token Test – Comprehension [Max = 21]	16.13	3.15	14.64	3.19	12.63	3.95	24.65**	0.16	1>2>3

* p<0.01, ** p<0.001.

Max: Maximum possible score on the test. NA: Normal Aging, MCI: Mild Cognitive Impairment, AD: Alzheimer's dementia, F =  Female, DEP  =  Depressed, RAVLT: Rey Auditory-Verbal Learning Test, IR: Immediate Recall, DR: Delayed Recall, Rec: Recognition, TN-LIN: Teste de Nomeação do Laboratório de Investigações Neuropsicológicas (*Naming Test* of the *Laboratory of Neuropsychological Investigations*).

The Neuropsychological battery formed four related factors (Table 2). The first factor was composed by tests related to verbal fluency, working memory and the Frontal Assessment Battery (*Executive Functions Factor)*. The second component contained the three TN-LIN variables, a test designed to assess the naming skills (*Language/Semantic Memory Factor)*. The third component contained the RAVLT items (*Episodic Memory Factor)*. The last component contained tests designed to assess visuospatial abilities, visual search (*Visuospatial Abilities Factor)*. Unexpectedly, a verbal comprehension test was also present in this fourth factor. These four factors explained 65% of the total variance. The internal consistency of the individual factors and the protocol as a whole was 0.83 satisfactory.

**Table 2:** Factor structure of the neuropsychological assessment protocol.

Neuropsychological Test	ExecutiveFunctions	Language/Semantic Memory	EpisodicMemory	VisuospatialAbilities
Letter Fluency (S)	**0.649**	0.099	−0.286	−0.070
Category Fluency (Animals)	**0.579**	0.035	−0.313	−0.141
Category Fluency (Fruits)	**0.503**	0.026	−0.318	−0.086
Frontal Assessment Battery	**0.435**	0.121	−0.126	0.288
Digit Span Forward	**0.426**	−0.018	0.064	0.115
Digit Span Backward	**0.417**	0.065	0.062	0.247
TN-LIN (Professions)	−0.021	**0.974**	0.016	−0.075
TN-LIN (Verbs)	−0.001	**0.839**	0.070	−0.017
TN-LIN (Nouns)	−0.023	**0.760**	−0.076	0.103
RAVLT (IR)	−0.006	−0.014	**−0.918**	−0.037
RAVLT (DR)	−0.033	0.054	**−0.910**	−0.030
RAVLT (Total)	0.070	0.011	**−0.810**	0.001
RAVLT (A1)	−0.015	−0.006	**−0.669**	0.133
RAVLT (Rec)	0.003	0.028	**−0.558**	0.189
Token Test – Comprehension	0.286	0.075	−0.041	**0.508**
Token Test – Attention	−0.013	0.046	−0.049	**0.491**
Stick Design Test	0.041	0.046	−0.128	**0.461**
Clock Drawing Test	**0.304**	0.135	−0.119	**0.318**
Eigenvalue	7.901	1.969	1.325	1.085
Variance Explained	42%	10%	7%	6%
Cronbach's Alpha	0.723	0.808	0.786	0.731

NA: Normal Aging, MCI: Mild Cognitive Impairment, AD: Alzheimer's dementia, RAVLT: Rey Auditory-Verbal Learning Test, IR: Immediate Recall, DR: Delayed Recall, Rec: Recognition, TN-LIN: Laboratory of neuropsychological Investigations Naming Test.

**Figure 1:**
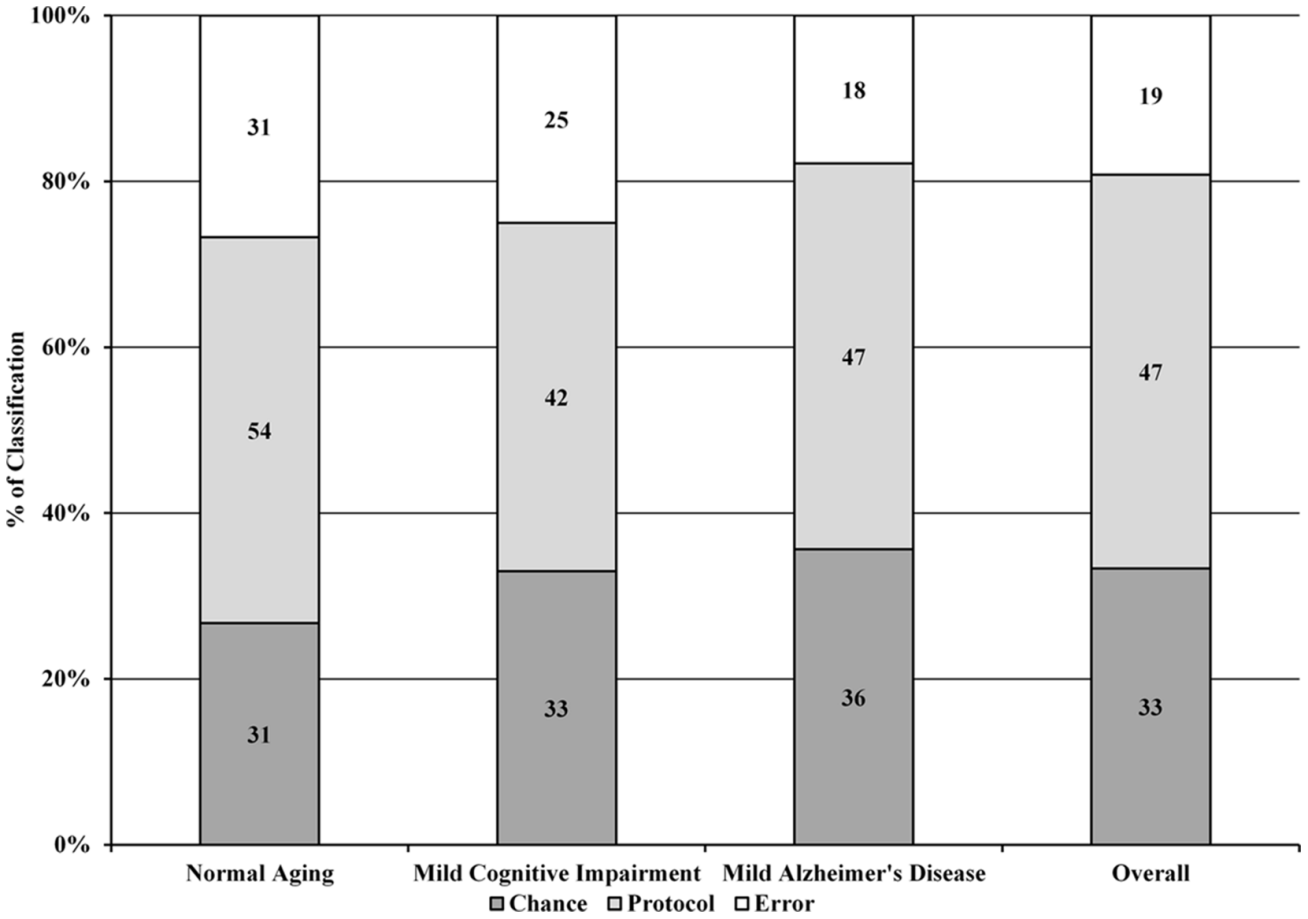
Stepwise linear regression model for the association between cognitive and functional measures. Legend: the four components extracted on the factor analysis (Episodic Memory, Executive Functions, Language/Semantic Memory and Visuospatial Abilities) were used as independent predictors of older adults performance on Activities of Daily Living. Three of the four components (excluding Visuospatial Abilities) explained about 30% of functional performance variance.

The ecological validity of the neuropsychological assessment was assessed by a stepwise linear regression model. The model was significant (F = 40.65, p<0.001, R^2^ = 31%) and contained three steps. The final model (third step) consisted of *Executive Functions* (β = 0.27, p<0.001), *Episodic Memory* (β = 0.20, p = 0.002) and *Language/Semantic Memory* (β = 0.23, p = 0.006), but not *Visuospatial* abilities (β = −0.11, p = 0.116). Figure 1 shows the relationship between the standardized predictors and the functional measure.

Table 3 shows the cut-off values for differentiating the diagnostic groups. All neuropsychological tests showed good sensitivity and specificity values to discriminate between NA and AD participants. Nonetheless, we observed that the sensitivity and specificity of the neuropsychological tests for discriminating NA vs. MCI, and MCI vs. AD were lower than those found for NA vs. AD.

**Table 3:** NA, MCI and AD non-depressed patients' description and ROC Curve Analysis.

Neuropsychological Tests	NA x AD				NA x MCI				MCI x AD			
	AUC (SE)	Cutoff	Sens.	Spec.	AUC (SE)	Cutoff	Sens.	Spec.	AUC (SE)	Cutoff	Sens.	Spec.
**Letter Fluency (S)**	0.85 (0.04)***	10	71%	77%	0.71 (0.05)***	11	65%	64%	0.65 (0.05)**	8	57%	41%
**Category Fluency (Animals)**	0.92 (0.02)***	12	81%	86%	0.75 (0.04)***	13	76%	67%	0.77 (0.04)***	10	70%	35%
**Category Fluency (Fruits)**	0.87 (0.03)***	11	74%	83%	0.79 (0.04)***	12	68%	79%	0.68 (0.05)***	9	57%	39%
**Frontal Assessment Battery**	0.91 (0.03)***	12	84%	80%	0.78 (0.04)***	14	71%	74%	0.75 (0.04)***	11	68%	26%
**Digit Span Forward**	0.69 (0.05)***	27	60%	59%	-	-	-	-	0.68 (0.05)***	27	64%	41%
**Digit Span Backward**	0.82 (0.04)***	11	76%	76%	0.67 (0.05)***	14	60%	71%	0.65 (0.05)**	9	65%	46%
**TN-LIN (Nouns)**	0.84 (0.04)***	26	74%	85%	0.62 (0.05)*	38	65%	60%	0.78 (0.04)***	36	71%	27%
**TN-LIN (Verbs)**	0.70 (0.05)***	9	86%	53%	-	-	-	-	0.67 (0.05)***	10	78%	49%
**TN-LIN (Professions)**	0.78 (0.04)***	14	79%	73%	0.67 (0.05)***	14	79%	56%	0.68 (0.05)***	13	73%	39%
**RAVLT (IR)**	0.93 (0.02)***	5	82%	86%	0.87 (0.03)***	6	73%	87%	0.62 (0.05)*	3	57%	42%
**RAVLT (DR)**	0.93 (0.02)***	5	82%	88%	0.87 (0.03)***	5	81%	71%	0.64 (0.05)***	2	67%	47%
**RAVLT (Total)**	0.91 (0.03)***	38	86%	82%	0.82 (0.04)***	32	74%	75%	0.67 (0.05)***	23	64%	41%
**RAVLT (A1)**	0.83 (0.04)***	4	79%	76%	0.75 (0.04)***	4	79%	60%	0.62 (0.05)*	4	40%	24%
**RAVLT (Rec)**	0.93 (0.02)***	5	82%	85%	0.88 (0.03)***	6	77%	81%	-	-	-	-
**Token Test – Comprehension**	0.84 (0.04)***	14	86%	79%	0.73 (0.05)***	15	69%	68%	0.68 (0.05)***	14	56%	32%
**Token Test – Attention**	0.68 (0.05)***	15	87%	49%	-	-	-	-	-	-	-	-
**Stick Design Test**	0.77 (0.04)***	11	94%	59%	0.64 (0.05)**	11	94%	33%	0.64 (0.05)**	11	67%	41%
**Clock Drawing Test**	0.87 (0.03)***	3	76%	85%	0.73 (0.05)***	4	76%	60%	0.68 (0.05)***	2	73%	46%

* p<0.05, ** p<0.01, *** p<0.001.

NA: Normal Aging, MCI: Mild Cognitive Impairment, AD: Alzheimer's Dementia, Med: Median, AUC: Area under the Curve, SE: Standard Error, Sens.: Sensibility, Spec.: Specificity, TN-LIN: Teste de Nomeação do Laboratório de Investigações Neuropsicológicas (*Naming Test* of the *Laboratory of Neuropsychological Investigations*), RAVLT: Rey Auditory-Verbal Learning Test, IR: Immediate Recall, DR: Delayed Recall, Rec: Recognition.

**Figure 2:**
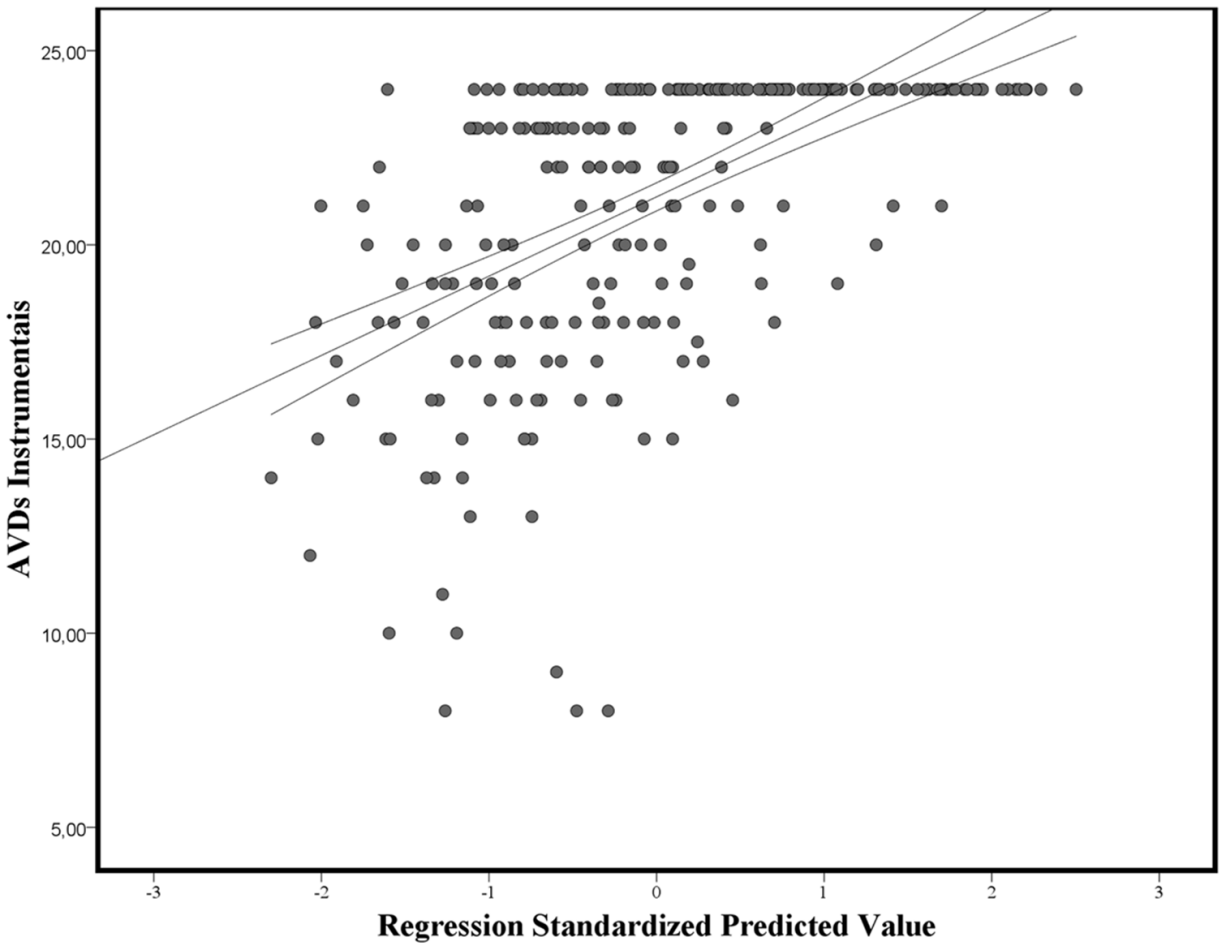
Stepwise multinomial regression models for the classification of the participants. Legend: increase on the classification rate of the participants by the use of different neuropsychological tests. The final model contains tests of general cognitive function (Mini-Mental State Examination), Episodic Memory (Delayed Recall and Recognition from the Rey Auditory-Verbal Learning Test), Executive Functions (Category Fluency Test “Animals” and the Frontal Assessment Battery) and Language/Semantic Memory (Laboratory of Neuropsychological Investigations Naming Test “Nouns”). Considering its general accuracy the protocol improves the classification rate of the participant's in 47%, starting at chance (33%). Its accuracy is greater for the identification of normal Aging (54%) followed by AD (47%) and MCI (42%), which reflect the intermediate condition of this last group.

The multinomial logistic regression model, which seeks to correctly classify the participants (considering the *Normal Aging* group as reference), was significant (−2 Log Likelihood  = 198.10, χ^2^ = 221.43, p<0.001, R^2^ = 78%). Six steps were performed by the model starting with RAVLT-DR (χ^2^ = 114.43, p<0.001), then adding, Mini-Mental State Examination (χ^2^ = 44.02, p<0.001), Category Fluency Animals (χ^2^ = 30.76, p<0.001), RAVLT-Rec (χ^2^ = 13.33, p<0.001), TN-LIN-Nouns (χ^2^ = 12.05, p = 0.002) and the Frontal Assessment Battery (χ^2^ = 6.83, p = 0.033). The participant's classification, considering chance, the final regression model and error are shown in Figure 2.

## Discussion

Our results suggest that this neuropsychological protocol has appropriate psychometric properties and can be used in the assessment older adults with low educational level. The factor analysis indicated a four component model related to Executive Functions, Language/Semantic Memory, Episodic Memory and Visuospatial Abilities, suggesting that the selected neuropsychological tests retain its construct validity for the assessment of older adults with low formal education. The structure is very similar to our a priori hypothesis, differing slightly on two neuropsychological measures. The neuropsychological tests performed well to differentiate healthy adults from MCI and AD patients and the performance on these tests were correlated to functional performance. These results indicate a good criterion related and ecologic validity of this neuropsychological battery.

The factor analysis showed a four factor structure for the neuropsychological battery (Executive Function, Language/Semantic memory, Episodic memory, and Visoespacial abilities). This is in accordance with the expected protocol structure given the tests chosen included in the neuropsychological protocol. Therefore, our results show that this protocol has good construct validity and is appropriate for administration in older adults with low educational level. This is particularly important as most of these tests were developed in countries with population with higher educational attainment level.

Three of the four cognitive components of our study correlated with functional performance and only visuospatial abilities were not correlated with functional performance. Previous studies, nonetheless, reported significant associations between visuospatial tests and functional measures [38], [39]. We delineate three hypotheses for the lack of significance. First, this factor has shown relatively low variance, since more pronounced visuospatial impairment is unlikely in normal aging, MCI and AD. The tests related to this factor are heterogeneous, and in all of them, there was a tendency for ceiling effects reducing the variance of this cognitive component. Finally, the IADLs assessed in this study are poorly related to spatial orientation, navigating, perception and spatial processing, with only one demanding a greater loading of spatial abilities (go out alone to distant locations using transport) [35]. These factors might have contributed to the lack of significant association between visoespacial abilities and functional performance. The analysis of the protocol ecological validity contributes for a topic usually neglected on the study of more traditional neuropsychological measures and might be useful from a prospective view, estimating environmental needs of the patients and guiding rehabilitation routines [22].

This neuropsychological protocol showed good criterion related validity, and the cutoff values found on this study could be used on clinical setting for discriminating AD, MCI and healthy older subjects. The best sensitivity and specificity values were observed for AD vs. healthy controls. The sensitivity and specificity for AD vs. MCI and MCI vs. NA was lower than those observed for AD vs. NA, but still at an acceptable range to be used in clinical practice. Our results are in accordance with other studies, with minor differences in the proposed cut-off value, and differences might be explained by sample particularities [2], [5], [10], [12]. Interestingly, our results are similar to those found in another Brazilian study which examined an elderly population with higher educational level and used a different neuropsychological protocol [2]. In this study, the authors also report that the best sensitivity and specificity values were observed for differentiating AD from healthy controls. The sensitivity and specificity for discriminating AD from MCI, and MCI from NA were lower. These results suggest that the identification of MCI and its differentiation from healthy older adults and early AD subjects are major challenges in clinical practice. The development of novel neuropsychological tests and protocols as well as the evaluation of combined tools and methods specifically designed for the diagnosis of MCI is necessary to improve our ability to make an accurate and early identification of these subjects.

The neuropsychological assessment protocol proposed by this study significantly improved the classification of the three groups. A multinomial regression model included tests of delayed and recognition memory (RAVLT), global cognitive functioning (Mini-Mental State Examination), executive functions (Category Fluency Animals and FAB) and language/semantic processing (TN-LIN Nouns). These results support the hypothesis that adding cognitive tests of different cognitive domains increase diagnostic power [40]. The accuracy, however, was lower than in other studies, such as Schmand and colleague [3]. Our analysis involves three different groups hypothetically defined as a continuum (NA – MCI – AD) and the cognitive boundaries of each one are largely superimposed. In a previous study, we found large effect sizes when AD patients and normal controls were compared using Token Test raw scores, however, when scaled scores based on population norms were adopted the effect sizes were only moderate [10]. We believe the proposed cutoff scores of our study might improve the clinical applicability of these measures on participants with low formal education.

Our study was performed with a very particular and vulnerable population, and the proposed cutoff scores might improve the clinical use of the neuropsychological testes for patients with similar characteristics. In this sense, the study is clinically relevant, with different cutoffs for several neuropsychological tests for three different comparisons, and could be used along with other methods to the diagnosis of AD and MCI, as well for the differentiation of these two conditions. However the cutoffs must be used cautiously, since the accuracy of each test independently is usually moderate, especially on the MCI x AD differentiation.

### Limitations

The present results should be viewed in light of study limitations. This is a cross-sectional study and the MCI patients were not followed-up to assess the progression to dementia. Previous studies suggest that there are significant baseline differences in cognitive performance between MCI converters and non-converters what may significantly influence the definition of cut-off scores to differentiate MCI from normal aging and dementia subjects [40], [41], [42]. The participants were recruited from a geriatric outpatient clinic and the present results may not be generalized to the general population. Additional studies with independent and community-based samples are necessary to evaluate and validate the psychometric properties and the proposed cut-off scores for the neuropsychological protocol.

## Conclusion

The present study shows strong evidence of the validity of a neuropsychological protocol designed for the cognitive assessment of older adults with low educational level. The measures are valid for the assessment of executive functions, language, memory and visuospatial abilities. It has good accuracy for the diagnosis of AD and MCI patients. Future studies are necessary to replicate these finding, to verify its applicability under other clinical conditions and to develop population-based norms for this protocol.
