# Correction: Clinical Applicability and Cutoff Values for an Unstructured Neuropsychological Assessment Protocol for Older Adults with Low Formal Education

**DOI:** 10.1371/annotation/2a374fe8-6d25-45cb-b97b-a5ca1529aa14

**Published:** 2013-10-01

**Authors:** Jonas Jardim de Paula, Laiss Bertola, Rafaela Teixeira Ávila, Lafaiete Moreira, Gabriel Coutinho, Edgar Nunes de Moraes, Maria Aparecida Camargos Bicalho, Rodrigo Nicolato, Breno Satler Diniz, Leandro Fernandes Malloy-Diniz

The titles and legends of Figures 1 and 2 were incorrectly switched. The title and legend currently appearing with Figure 1 belong with Figure 2, and the title and legend currently appearing with Figure 2 belong with Figure 1. The Figures are in correct order. 

